# Clinical Tests of Hearing—II

**Published:** 1909-09-18

**Authors:** 


### Otology.

### CLINICAL TESTS OF HEARING—II.

We now come to those tests designed to enable us to distinguish between defects of the perceptive apparatus and those of the conducting mechanism. The facts upon which these tests depend are very simple. A steadily diminishing sound, such as that produced by a vibrating tuning fork, is heard more loudly, and therefore longer, by aërial than by bone conduction, provided that the conducting mechanism is normal—that is, that the mobility of the drum-membrane and ossicles is unimpaired and that there is no obstruction in the tympanum or external auditory meatus. Any impairment of the conducting mechanism not only diminishes the conduction of sound through the air, but also increases the intensity of its conduction through the bones of the head. This may easily be proved by holding the base of a $C^2$ fork against the mastoid process until it ceases to be heard, when the sound will reappear on firmly stopping the ear with the finger. On the other hand, when deafness is due to disease of the perceptive apparatus, whether the lesion be in the labyrinth, auditory nerve, or cerebral centres, the bone conduction is reduced with the aërial conduction, and the tuning fork is heard longer through the air than over the mastoid process.

Rinne's test is simply the comparison of the air conduction with bone conduction. The base of the tuning fork is applied to the bone behind and slightly above the ear—that is, just over the antrum—until it ceases to be heard, when it is brought opposite the meatus; if the sound now reappears, Rinne's test is said to be *positive* ($R$ +). If it is not re-heard, the test is repeated in the opposite way, the fork being held near the meatus until the sound ceases, and then being applied behind the ear; if the sound is now again perceived, Rinne's test is *negative* ($R$ −). Although a "negative Rinne" points to disease of the conducting apparatus, and a "positive Rinne" to an affection of the perceptive mechanism, the matter is in reality not quite so simple as this. As Rinne is positive in a normal ear, it is obvious that there must be slight degrees of deafness due to lesions of the conducting apparatus in which a positive Rinne is still present. Supposing that the length of time during which the fork is normally heard by the air *after* it has ceased on the mastoid is 45 seconds, as in a $C^0$ fork of the writer's, Rinne will only become negative when the decrease of air conduction *plus* the increase of bone conduction together exceed 45 seconds. Again, the relative duration of the sound through the bone to that through the air varies with forks of different pitch when tested on a normal ear, the bone conduction being proportionately longer with low-pitched than with high-pitched forks; for instance, in a set belonging to the writer, the duration of bone conduction compared to air conduction is for the $C^0$ fork 1 : 2, for the $C^2$ fork 1 : 3, and for the $C^4$ fork 1 : 5. Therefore Rinne's test will more readily become negative in the lower part of the scale, and, especially in slight degrees of deafness, the test should be made with the $C^0$ fork. Again, in cases of severe deafness, involvement of the internal ear can only be excluded if Rinne is negative with the $C^2$ as well as the $C^0$ fork; for when severe deafness is caused entirely by disease of the conducting mechanism, Rinne's test is negative up to or above this point of the scale. In severe unilateral nerve-deafness the fork on the mastoid may be heard by the opposite ear, and Rinne's test be thus incorrectly taken as negative.

Schwabach's test is the comparison between the bone conduction of the patient's ear and that of the examiner. If the latter is not normal, the abnormality must of course be allowed for. The test is made by transferring the fork from the examiner's to the patient's mastoid, or *vice versa*, as soon as the sound has ceased and noting the number of seconds above or below normal over which the patient hears. The test is mentioned here by name for the sake of clearness, as in Continental writings it often appears as "Schwabach +" or "Schwabach −"; but the multiplication of names is to be deprecated, and English authors usually write the result simply as "bone conduction +" or "B.C. + 12″." It is wise to make this test both with the $C^0$ and $C^2$ forks, and it forms a valuable check to Rinne's test. Increase of bone conduction means disease of the conducting apparatus, and is the natural accompaniment of a negative Rinne; but in old-standing cases of middle-ear disease, and especially in otosclerosis, the bone conduction is often diminished a little, but slight diminution (*i.e.* under 10 seconds) need not be taken to imply disease of the perceptive apparatus.

Weber's test is the comparison of the bone conduction of the patient's two ears. The fork is placed on the middle line of the forehead or vertex, and the patient is asked to state on which side the sound is louder. It is obviously only of value where one ear is much deafer than the other. In middle-ear deafness the sound is better heard in the deafer ear, whilst in nerve-deafness it sounds louder in the good ear. It is useful in cases of extreme unilateral deafness where Rinne's and Schwabach's tests are open to error owing to conduction of sound to the good ear.

Gellé's test is more rarely required; its object is to diagnose fixation of the stapes, and it depends on the fact that increased labyrinthine pressure diminishes the perception of sound. A fork is placed against the mastoid and the air in the meatus is compressed by a Siegle's speculum attached to a rubber ball. If during the compression the fork is heard less well, the stapes is obviously movable and Gellé's test is said to be positive; if the sound is unaffected by compression, ankylosis of the stapes is present and Gellé's test is negative. The stapes becomes fixed in late stages of otosclerosis and chronic catarrh, and in such cases Rinne should be negative up to or above $C^2$; but if there be, as often happens, secondary involvement of the internal ear, Rinne may be positive at $C^2$ and negative at $C^0$; here the Gellé's test will decide the question of stapes ankylosis.

